# The Etiology and Antimicrobial Susceptibility of Community-Onset Urinary Tract Infections in a Low-Resource/High-Resistance Area of Latin America

**DOI:** 10.3390/tropicalmed10030064

**Published:** 2025-02-27

**Authors:** Maria Micieli, Selene Rebecca Boncompagni, Tiziana Di Maggio, Yenny Bertha Mamani Ramos, Antonia Mantella, Ana Liz Villagrán, Carmen Angélica Revollo Yelma, Evelin Esther Fortún Fernández, Michele Spinicci, Marianne Strohmeyer, Lucia Pallecchi, Gian Maria Rossolini, Alessandro Bartoloni

**Affiliations:** 1 Department of Experimental and Clinical Medicine, University of Florence, 50134 Florence, Italy; maria.micieli@unifi.it (M.M.); selenerebecca.boncompagni@unifi.it (S.R.B.); antoniamantella@libero.it (A.M.); michele.spinicci@unifi.it (M.S.); marianne.strohmeyer@unifi.it (M.S.); gianmaria.rossolini@unifi.it (G.M.R.); 2 Department of Medical Biotechnologies, University of Siena, 53100 Siena, Italy; tiziana.dimaggio@unisi.it (T.D.M.); lucia.pallecchi@unisi.it (L.P.); 3 Hospital Básico Villa Montes, Villa Montes 00591, Bolivia; yennymamaniramos@gmail.com (Y.B.M.R.); analiz76385796@gmail.com (A.L.V.); 4 Instituto Nacional de Laboratorios de Salud “Dr. Nestor Morales Villazón” (INLASA), La Paz 00201, Bolivia; yelmacar@yahoo.com (C.A.R.Y.); evfortun@minsalud.gob.bo (E.E.F.F.); 5 Infectious and Tropical Diseases Unit, Careggi University Hospital, 50134 Florence, Italy; 6 Microbiology and Virology Unit, Florence Careggi University Hospital, 50134 Florence, Italy

**Keywords:** Bolivia, community-onset urinary tract infections, MDR, empirical therapy, *Escherichia coli*

## Abstract

Urinary tract infections (UTIs) are common and are typically treated empirically, based on local antimicrobial resistance (AMR) data, which are often scarce in low- and middle-income countries. This study examines the AMR patterns of pathogens causing community-onset (CO) UTIs in the Bolivian Chaco. Urine samples were collected from subjects with suspected CO-UTIs and analyzed by culture techniques. Significant isolates were tested for their antimicrobial susceptibility. Additionally, *bla*_CTX-M_ and *mcr* genes were searched for using real-time PCR. A total of 361 CO-UTI episodes were diagnosed among 731 subjects from February 2020 to November 2021. The cases included uncomplicated and complicated UTIs (58.2% and 41.8%, respectively), with females accounting for the majority (85.3%) of cases. *Escherichia coli* was the most prevalent pathogen (86.6%), followed by *Klebsiella pneumoniae* (5.4%) and *Proteus* spp. (2.2%). Very high resistance rates (>50%) were observed for ampicillin, trimethoprim–sulfamethoxazole and fluoroquinolones, high resistance rates (>20%) for amoxicillin–clavulanate, third-generation cephalosporins and gentamicin, while lower resistance rates (<10%) were observed for nitrofurantoin and fosfomycin. The prevalence of *bla*_CTX-M_ among *E. coli* was high (26.7%). Colistin resistance was detected in 3.4% of *E. coli*, mostly associated with *mcr* genes. CO-UTIs from this area were characterized by high resistance rates to commonly used antibiotics (trimethoprim–sulfamethoxazole, amoxicillin–clavulanic acid and ciprofloxacin), highlighting the importance of knowledge of the local epidemiology to inform the selection of appropriate empirical antibiotic regimens.

## 1. Introduction

Urinary tract infections (UTIs) are among the most common community- and hospital-acquired bacterial infections [1]. UTIs are classified as uncomplicated (uUTI) or complicated (cUTI), with the former occurring predominantly in non-pregnant women in the absence of functional or anatomical abnormalities or co-morbidities, while the latter is typically associated with risk factors that compromise either urodynamics or host defenses, such as indwelling or intermittent urinary catheterization, urinary obstruction or retention, immunosuppression, renal failure, renal transplantation, diabetes, male sex and pregnancy [1,2,3].

The diagnosis of uUTIs is usually based on a clinical history of lower urinary tract symptoms (dysuria, frequency and urgency) in the absence of known risk factors for cUTIs, while a urine culture is mostly recommended in patients with atypical symptoms and in those who fail to respond to empiric antimicrobial therapy [3]. Consequently, treatment for uUTIs is usually prescribed on an empirical basis, considering the local epidemiology of antimicrobial susceptibility among the most common pathogens (e.g., *Escherichia coli* and other Enterobacterales, and *Staphylococcus saprophyticus*) [1,4,5,6].

In low- and middle-income settings, where access to healthcare facilities is often challenging and diagnostic facilities may be unavailable, epidemiological data on the antimicrobial susceptibility of pathogens that are responsible for UTIs are often missing [7], and the rates of antimicrobial resistance among UTI pathogens can be substantially different from that observed in higher-income settings and empiric regimens designed on epidemiological data deduced from other settings may be misleading. For this reason, a broader knowledge of the epidemiology of UTIs in low- and middle-income settings is warranted.

Here, we report the results of an investigation on the epidemiology and antimicrobial susceptibility of major bacterial pathogens that are responsible for community-onset (CO) UTIs from an urban area in the Bolivian Chaco.

## 2. Materials and Methods

### 2.1. Study Design

A cross-sectional study was carried out from February 2020 to November 2021 in a small urban area in the Bolivian Chaco (Villa Montes, Tarija Department, Plurinational State of Bolivia). This study enrolled adult subjects experiencing symptoms suggestive of urinary tract infection (dysuria, frequency and urgency) [3,8], who sought medical care at primary health centers present in Villa Montes, or who requested consultation as outpatients at hospitals in the same area. A convenience sampling method was used, including all adult patients who consented to participate in this study during the 22-month enrollment period. This study was preceded by an information event involving local general practitioners, pharmacists and laboratory personnel. During the study period, urine culture and antimicrobial susceptibility analysis were offered free of charge. Patients were advised to provide urine samples before initiating any prescribed antibiotic therapy. From all enrolled patients, demographic data and a brief medical history were collected through a questionnaire. A first morning mid-stream urine specimen was collected from each enrolled patient in a sterile container without boric acid, stored at 4 °C and transferred to the local laboratory within 4 h. Each specimen was investigated by cultural analysis as detailed below.

A case was defined when cultural analysis confirmed the presence of one or two pathogens with a bacterial count >10^5^ CFU/mL [9]. To focus this study on non-recurrent UTIs, when a subject yielded two or more positive urine samples during the study period, only samples collected at least 6 months apart and/or separated by at least one negative urine sample were included in the analysis as independent cases [3]. The microbiologically confirmed CO-UTIs were classified as complicated or uncomplicated based on the medical history of the enrolled subjects, according to the European Association of Urology (EAU) and the Bolivian Health Authority (Autoridad de Supervisión de la Seguridad Social de Corto Plazo—ASUSS) guidelines [3,8]. In particular, a case was defined as uUTI in the absence of known anatomical abnormalities, comorbidities or risk factors. In contrast, cUTI was defined when risk factors, such as male sex, urinary catheterization, obstruction, immunosuppression, renal failure, diabetes, or pregnancy were present [3,8]. Results of urine sample analysis were reported to patients and doctors. Evaluation of clinical and microbiological efficacy, and of appropriateness of the prescribed treatment, was beyond the scope of this study.

### 2.2. Bacterial Isolation, Identification and Antimicrobial Susceptibility Testing

At the laboratory of the Hospital Básico Villa Montes, each urine sample (10 µL) was cultured at 35 ± 2 °C, aerobically, for 18–24 h on cystine lactose electrolyte-deficient (CLED) and MacConkey agar media (Oxoid, La Paz, Bolivia). When bacterial growth occurred and was considered significant (>1 × 10^5^ CFU/mL), identification by standard microbiological procedures was performed (involving colonial morphology, Gram staining and biochemical tests) [9]. When identification returned major uropathogens (i.e., *E. coli*, *Klebsiella pneumoniae* or *Proteus* spp.), antimicrobial susceptibility testing was performed by disk diffusion on Mueller–Hinton agar (Oxoid, La Paz, Bolivia) according to standards from Clinical and Laboratory Standards Institute (CLSI) [10], and results were interpreted according to the CLSI clinical breakpoints [11]. Multi-drug resistant (MDR) phenotypes were defined in case of resistance to at least three different classes of antimicrobial agents [12]. The following antimicrobial agents (disk potency) were tested: ampicillin (10 µg), amoxicillin–clavulanic acid (20/10 µg), cefotaxime (30 µg), ceftazidime (30 µg), ciprofloxacin (5 µg), gentamicin (10 µg), imipenem (10 µg), meropenem (10 µg), nalidixic acid (30 µg), nitrofurantoin (300 µg) and trimethoprim–sulfamethoxazole (1.25/23.75 µg) (Oxoid, La Paz, Bolivia).

All isolates associated with an episode of CO-UTI were stored in Amies transport medium (Oxoid, Milan, Italy) at 4 °C, and were transferred to a secondary laboratory in Italy (Microbiology Laboratory of the Department of Experimental and Clinical Medicine, University of Florence, Florence, Italy) for further characterization. In this laboratory, identification of bacterial isolates was confirmed by MALDI-ToF mass spectrometry (Bruker Daltonics, Germany; MBT reference library, version 2022), and antimicrobial susceptibility to fosfomycin and colistin was tested by agar dilution and broth microdilution methods, respectively, in accordance with CLSI standards [10], for all confirmed *E. coli* isolates. In addition, screening for extended-spectrum β-lactamase (ESBL) production was carried out using the double-disk method with amoxicillin–clavulanate and cefotaxime [13] for all confirmed *E. coli* isolates showing a resistance profile, including expanded-spectrum cephalosporins.

### 2.3. Molecular Characterization of Acquired Resistance Genes

In all ESBL-positive isolates, screening for *bla*_CTX-M_ genes of different groups was performed using multiplex real-time polymerase chain reaction (mRT-PCR), as previously described [14]. Moreover, the presence of known acquired colistin resistance genes (*mcr-1-9*) was investigated in colistin-resistant *E. coli* isolates using mRT-PCR, as previously described [15,16]. Primers, probes and reaction conditions used for the mRT-PCR assays are detailed in Tables S1 and S2.

### 2.4. Statistical Analysis

Statistical analysis was performed using Fisher’s exact test, using R version 3.2.0 for Windows. A *p*-value of <0.05 was interpreted as statistically significant.

## 3. Results

### 3.1. Antibiotic Prescribing Patterns in Uncomplicated and Complicated Community-Onset Urinary Tract Infections

**Figure 1:**
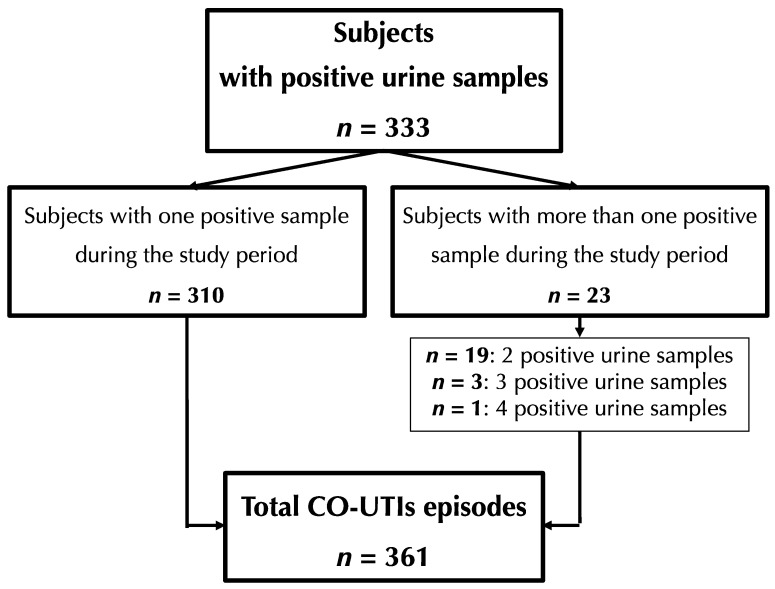
Community-onset urinary tract infection episodes, Villa Montes, Bolivia, 2020–2021.

A total of 731 adults (median age of 41, IQR = 28; male–female ratio of approx. 1:4) experiencing symptoms of UTI were included in this study. Overall, 333 of them (45.6%) yielded one (*n* = 310) or more (*n* = 23) positive urine cultures that were considered consistent with the case definition, leading to a total of 361 episodes of CO-UTIs, with females accounting for the majority (85.3%) of cases (Figure 1). Among the 361 cases, 210 (58.2%) were classified as uUTIs (all in females), while the remaining 151 were classified as cUTIs, including 60 (39.7%) non-pregnant women, 38 (25.2%) pregnant women and 53 (35.1%) males (Table 1). The age distribution varied by gender, with the majority of male patients being 65 years or older (males ≥ 65, *n* = 30/53, 56.6%; females ≥ 65, *n* = 53/308, 17.2%; *p* < 0.05).

Information about empirically prescribed antibiotics, which was available for 149 individuals, revealed that fluoroquinolones (e.g., ciprofloxacin, norfloxacin or levofloxacin) were the most commonly prescribed antibiotics (48%), followed by nitrofurantoin (20%), amoxicillin (11%), trimethoprim–sulfamethoxazole (6%), third-generation cephalosporins (cefotaxime or cefixime) (5%), aminoglycosides (4%), amoxicillin–clavulanate (3%) and other drugs (3%). Notably, no one reported being prescribed fosfomycin.

### 3.2. Etiology and Antimicrobial Resistance of Community-Onset Urinary Tract Infections

Of the 361 cases of microbiologically confirmed CO-UTIs, 350 (97%) were positive for a single pathogen and 11 (3%) for two pathogens, yielding a total of 372 bacterial isolates. The most frequently isolated species was *E. coli* (86.4%), followed by *K. pneumoniae* (5.4%) and *Proteus mirabilis* (1.6%). Other Enterobacterales, Gram-negative nonfermenters, *S. saprophyticus* and *Enterococcus faecalis* accounted for only a minority of cases, with no significant differences between uUTIs and cUTIs (Table 2). The etiology of CO-UTIs stratified by patient group is presented in Supplementary Table S3.

Bacterial identification at the secondary laboratory was concordant with that reported by the local laboratory in 90.3% of all cases. Most discrepancies (31 of 35) occurred at the species or genus level within the order Enterobacterales, while four isolates that were identified initially as *Serratia marcescens* were re-identified as Gram-negative nonfermenters (Supplemental Table S4).

**Figure 2:**
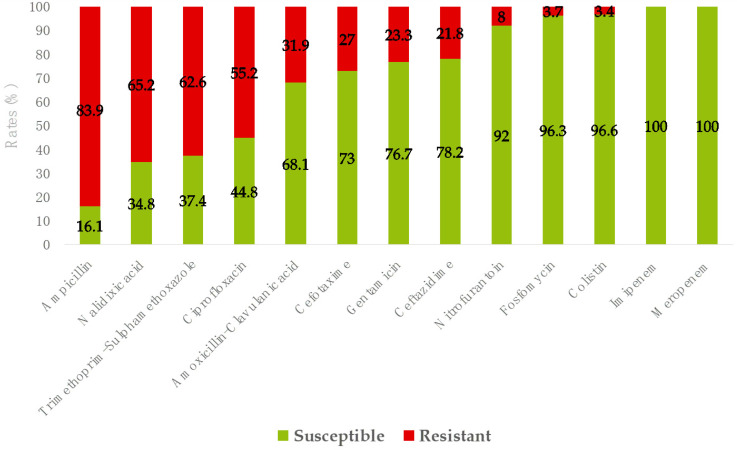
Antibiotic susceptibility of *E. coli* (*n* = 322), *K. pneumoniae* (*n* = 20) and *P. mirabilis* (*n* = 6) from community-onset urinary tract infections in Villa Montes, Bolivia, 2020–2021. Resistance rates to ampicillin did not include *K. pneumoniae* isolates. Antibiotic susceptibility rates for colistin and fosfomycin were determined only in *E. coli*.

Considering *E. coli*, *K. pneumoniae* and *P. mirabilis*, which were the most common pathogens, very high resistance rates (>50%) were observed for ampicillin, trimethoprim–sulfamethoxazole, nalidixic acid and ciprofloxacin, and notably high resistance rates (>20%) were observed for amoxicillin–clavulanate, third generation cephalosporins and gentamicin, while a resistance rate <10% was observed for nitrofurantoin, and no resistance was observed for carbapenems (Figure 2). As for *E. coli*, resistance rates to fosfomycin and colistin, determined by reference methods, were 3.7% and 3.4%, respectively (Figure 2).

No significant differences were observed in the resistance rates of isolates from uUTIs and cUTIs (Table S5).

With respect to *E. coli*, the most represented pathogen, we observed statistically significant differences in antimicrobial susceptibility among different patient groups (females with uUTIs, males, pregnant women, females with cUTI, subjects with history of recurrent UTI and subjects with a history of previous UTIs). Specifically, the following were identified: (a) lower susceptibility rates to ampicillin, trimethoprim–sulfamethoxazole, ciprofloxacin, cefotaxime, ceftazidime and gentamicin were observed in males compared to females (Figure S1); (b) a lower susceptibility rate to nitrofurantoin was observed in subjects with a history of recurrent UTIs (Figure S2); and (c) higher susceptibility rates to trimethoprim–sulfamethoxazole, cefotaxime and ceftazidime were observed in pregnant women (Figure S2).

Moreover, the majority of pathogens (191/348, 54.8%) showed an MDR profile, with *E. coli* being the predominant one (*n* = 182, 95.3%). A significantly higher prevalence of MDR was observed in males compared to females (*n* = 36/50, 72% vs. *n* = 53/298, 52.3%, respectively; *p* = 0.01).

The most common co-resistance patterns among MDR *E. coli* were to beta-lactams, quinolones/fluoroquinolones and folate pathway inhibitors (*n* = 86/182, 47.3%), and to aminoglycosides, beta-lactams, quinolones/fluoroquinolones and folate pathway inhibitors (*n* = 55/182, 30.2%). The complete list of co-resistance patterns among the MDR isolates is reported in Table S6.

### 3.3. Molecular Characterization of Acquired Resistance Genes Among E. coli

Regarding resistance mechanisms, all *E. coli* isolates that were resistant to cefotaxime (*n* = 91) were confirmed as ESBL producers by phenotypic testing, and most of them (*n* = 86/91, 94.5%) were found to carry *bla*_CTX-M_ genes using molecular testing. A significant difference in the prevalence of *bla*_CTX-M_ genes among total *E. coli* isolates was observed between males and females (males: 21/43, 48.8%; females: 65/213, 30.5%; *p* = 0.001). The CTX-M-1-group was the most prevalent (*n* = 54/86, 62.8%), followed by the CTX-M-9-group (*n* = 32/86, 37.2%). CTX-M-positive *E. coli* were significantly more resistant than CTX-M-negative *E. coli* to quinolones, trimethoprim–sulfamethoxazole, amoxicillin–clavulanate, colistin and fosfomycin, while no significant differences were observed between the two groups for nitrofurantoin (Supplemental Table S7).

With respect to colistin resistance, all but one of the colistin-resistant *E. coli* isolates (*n* = 10/11, 90.9%) were found to carry the *mcr-1* like genes (all from female patients).

## 4. Discussion

UTIs are among the most common infections in primary care and are a major driver for prescribing antibiotics. CO-uUTIs, which occur in otherwise healthy individuals outside of hospital settings, are usually treated empirically [16], based on the prescribers’ experience and behavior. Therefore, knowledge of the antimicrobial resistance epidemiology among uropathogens in specific areas is crucial for determining the most appropriate empirical antimicrobial treatment. However, when available, data from diagnostic microbiology laboratories may be subject to selection bias due to the over sampling of clinical specimens from patients with initial treatment failures, complicated clinical histories or severe infections. This sampling bias may lead to overestimating the burden of antimicrobial resistance in the community setting.

We carried out this study in Villa Montes, a small city in the Bolivian Chaco, to fill this crucial knowledge gap with respect to antimicrobial resistance in low- and middle-income settings, where structured surveillance systems and diagnostic networks are often lacking, and to support prescribers to improve the management of CO-UTIs through an appropriate empirical treatment.

As far as the etiology is concerned, as expected, *E. coli* was the predominant pathogen, followed by *K. pneumoniae* and *P. mirabilis*. The use of mass spectrometry, in addition to biochemical tests, allowed the identification of some rarely encountered uropathogens.

In Bolivia, guidelines for the empiric treatment of uUTI recommend nitrofurantoin and fosfomycin as first-line therapeutic choices, with trimethoprim–sulfamethoxazole as a possible alternative if local resistance rates are <20%, and ciprofloxacin, amoxicillin–clavulanic acid and cephalosporins (e.g., cefixime, cefpodoxime and cefalexin) as second-line alternatives [8]. Previous studies conducted by our group in the same area showed relatively high resistance rates to some first- and second-line agents (e.g., fluoroquinolones, trimethoprim–sulfamethoxazole and third-generation cephalosporins) among commensal Enterobacterales from children and clinical isolates from a selected population, including inpatients and outpatients whose clinical specimens were analyzed at the clinical microbiology laboratory of the Villa Montes Hospital [17,18,19].

The present study revealed a remarkable prevalence of resistance among pathogens responsible for CO-UTIs in the Bolivian Chaco. Very high rates of resistance to ampicillin, trimethoprim–sulfamethoxazole and fluoroquinolones were observed. Furthermore, the dissemination of ESBL determinants among *E. coli* was notably high and linked to increased resistance to multiple antibiotics, including first- and second-line non-β-lactam agents (e.g., fosfomycin, trimethoprim–sulfamethoxazole and ciprofloxacin). This underscores the spread of MDR bacteria in the region and the challenges in selecting empirical antibiotic treatments. Importantly, our study did not identify any resistance to carbapenems in the analyzed strains. This finding is significant, as it highlights that carbapenem resistance may not yet be widespread in this region, contrasting with patterns observed in other settings. While this study highlights important resistance patterns, it does not address the mechanisms driving resistance. Future research could focus on phylogenetic and genomic studies to better understand the spread of resistant strains and genes, supporting more effective interventions.

Given the substantial resistance to other antibiotics, nitrofurantoin and fosfomycin emerged as the primary treatment options for uncomplicated UTIs. However, the fosfomycin resistance rate of 14% observed among ESBL producers should be a matter of concern, suggesting that additional drivers can influence acquired resistance to this drug in this region. In this perspective, the investigation of fosfomycin resistance mechanisms among pathogenic and commensal *E. coli* from this area could be of interest to better understand the evolution of this phenomenon. On the other hand, due to the very high resistance rates (63%), trimethoprim–sulfamethoxazole should not be considered a suitable alternative to first-line drugs in this setting.

Moreover, our findings indicate that fluoroquinolones, despite not being first-line agents and showing the highest resistance rates, were the most prescribed antibiotics for CO-UTIs, raising concerns about treatment outcomes.

Regarding the gender comparison, our study provided evidence of a significantly lower susceptibility rate for several antibiotics and a higher prevalence of both phenotypes and ESBL determinants among *E. coli* isolated from males. Since the majority of our male population was over 65 years old, these findings could be linked to the frequent association of UTIs with urological abnormalities, such as prostate involvement, which complicate the infection [20]. The prostate’s role as a parenchymatous organ often leads to more challenging eradication of bacteria, resulting in prolonged or recurrent antibiotic use [20]. Moreover, pregnant women exhibited better susceptibility patterns with significantly higher susceptibility to trimethoprim–sulfamethoxazole (not indicated in pregnancy), cefotaxime and ceftazidime compared to other patients. Finally, the group of patients with a history of recurrent UTIs exhibited significantly lower susceptibility to nitrofurantoin (13.1%) compared to that found in the remaining patients (8%).

Interestingly, the urinary *E. coli* isolates examined in this study demonstrated a comparable prevalence of CTX-M producers (26.7% vs. 27.9%, *p* = 0.9) and a similar distribution of CTX-M groups as those found among commensal *E. coli* collected in 2019 from healthy children from the same area [18]. These findings suggest that commensal bacteria could act as a crucial reservoir for resistant strains with the potential to behave as pathogens and for resistance genes that are transferable to pathogenic strains, underscoring the notion that commensal *E. coli* could serve as a valuable proxy in predicting the dissemination of resistance among pathogens [19,21].

Regarding colistin resistance, a study conducted in 2016 in the same area detected a 38.3% prevalence of *mcr-1* carriage among commensal strains from healthy children [22]. Considering that the use of colistin in Bolivia was occasional and limited to infections caused by some MDR pathogens in large urban hospitals, the unrestricted use of colistin in veterinary medicine and animal breeding and the importation of *mcr-1*-positive bacteria via food and animals were hypothesized to be the driving factors for the high prevalence of *mcr-1* carriage [22]. In 2019, a formal administrative resolution (N° 158/2019) from the Servicio Nacional de Sanidad Agropecuaria e Inocuidad Alimentaria (SENASAG) banned the use of colistin as a growth promoter (feed additive) [23]. Although colistin is not used for treating UTIs, and the resistance rates to colistin detected in the present study were relatively low, the finding of *mcr-1* in 3.1% of *E. coli* from CO-UTIs indicates the pervasiveness of these resistance determinants across strains circulating in the human population, emphasizing the need for continued monitoring and research.

There were some limitations in our study. The use of a bacterial count >10^5^ CFU/mL to define a positive urine culture is known to not be sensitive enough in certain cases of complicated UTIs, and using this criterion might have led to an underestimation of the positive cases. Additionally, the results reflect the epidemiological scenario of the population living in Villa Montes and may not be representative of other areas of the country. Finally, information on the prescription of empirical treatment was collected from the patients and not from the prescribers and covered only a limited number of patients.

## 5. Conclusions

This study provides, for the first time, some valuable insight into the etiology of CO-UTIs and the antimicrobial resistance patterns of causative uropathogens in an urban area in the Bolivian Chaco, filling a relevant epidemiological knowledge gap. The obtained results will be useful to guide clinicians to prescribe appropriate empiric treatment for CO-UTIs, minimizing the risk of ineffective therapy and reducing the misuse of antibiotics in the community. Similar studies are recommended to ensure that health care providers are informed about evolving resistance trends and to inform public health interventions.

## Figures and Tables

**Table 1:** Characteristics of subjects with community-onset urinary tract infections, Villa Montes, Bolivia, 2020–2021.

	Positive Urine Culture		
	Total (*n* = 361)	Uncomplicated (*n* = 210)	Complicated (*n* = 151)
Age: median (IQR)	38.5 (30)	42 (25)	55 (35)
Age groups, years			
18–64	278 (77)	175 (83.3) *	103 (68.2)
≥65	83 (23)	35 (16.7)	48 (31.8) *
Gender			
Females	308 (65.3)	210 (100)	98 (64.9)
Males	53 (14.7)	NA	53 (35.1)
Underlying diseases			
Renal failure	7 (1.9)	NA	7 (4.6)
Liver cirrhosis	1 (0.3)	NA	1 (0.7)
Diabetes mellitus	60 (16.6)	NA	60 (39.7)
Predisposing factors			
Previous UTI	67 (18.6)	35 (16.7)	32 (21.2)
Recurrent UTI	61 (16.9)	32 (15.2)	29 (19.2)
Catheter	11 (3)	NA	11 (7.3)
Pregnancy	38 (10.5)	NA	38 (25.2)
Prostatitis	5 (1.4)	NA	5 (3.3)

SD, standard deviation; IQR, interquartile range; NA, not applicable as underlying diseases and predisposing factors defining cUTI. *, *p* < 0.01 uUTI vs. cUTI; results are *n* (%) unless otherwise specified.

**Table 2:** Etiology of uncomplicated (uUTIs) and complicated (cUTIs) community-onset urinary tract infections in Villa Montes, Bolivia, 2020–2021.

	uUTIs (*n* = 213)		cUTIs (*n* = 159)		Total UTIs (*n* = 372)		*p* Value ^1^
Species	No.	%	No.	%	No.	%	
*Escherichia coli*	184	86.4	138	86.8	322	86.6	1
*Klebsiella pneumoniae*	10	4.7	10	6.3	20	5.4	0.5
*Proteus mirabilis*	4	1.9	2	1.3	6	1.6	1
*Morganella morganii*	2	0.9	2	1.3	4	1.1	1
*Citrobacter koseri*	2	0.9	-	-	2	0.5	-
*Staphylococcus saprophyticus*	2	0.9	-	-	2	0.5	-
*Acinetobacter junii*	1	0.5	-	-	1	0.3	-
*Acinetobacter pitti*	1	0.5	-	-	1	0.3	-
*Pseudomonas mendocina*	1	0.5	-	-	1	0.3	-
*Pseudomonas stutzeri*	1	0.5	-	-	1	0.3	-
*Enterobacter cloacae*	1	0.5	-	-	1	0.3	-
*Escherichia vulneris*	1	0.5	-	-	1	0.3	-
*Klebsiella variicola*	1	0.5	-	-	1	0.3	-
*Salmonella* spp.	1	0.5	-	-	1	0.3	-
*Stenotrophomonas maltophilia*	1	0.5	-	-	1	0.3	-
*Proteus vulgaris*	-	-	2	1.3	2	0.9	-
*Klebsiella oxytoca*	-	-	1	0.6	1	0.3	-
*Citrobacter freundii*	-	-	1	0.6	1	0.3	-
*Enterobacter asburiae*	-	-	1	0.6	1	0.3	-
*Enterococcus faecalis*	-	-	1	0.6	1	0.3	-
*Serratia marcescens*	-	-	1	0.6	1	0.3	-

^1^, calculated using Fisher’s exact test; *p* < 0.05, significant.

## Data Availability

The original contributions presented in this study are included in the article/Supplementary Material. Further inquiries can be directed to the corresponding author.

## Supplementary Materials

The following supporting information can be downloaded at the following: https://www.mdpi.com/article/10.3390/tropicalmed10030064/s1, References [24,25,26,27,28,29,30,31] are cited in the supplementary materials. Table S1: Primers and probes used in ESBL-positive *Escherichia coli* from community-onset urinary tract infections in Villa Montes, Bolivia, 2020–2021.; Table S2: Primers and probes used in colistin-resistant *E. coli* from community-onset urinary tract infections in Villa Montes, Bolivia, 2020–2021; Table S3: Etiology of community-onset urinary tract infections (UTI) stratified by patient group, in Villa Montes, Bolivia, 2020–2021; Table S4: Discrepancies in bacterial identification between local laboratory (Bolivia) and secondary laboratory (Italy); Table S5. Antibiotic susceptibility rates (%) of *E. coli* (*n* = 322), *K. pneumoniae* (*n* = 20) and *Proteus mirabilis* (*n* = 6) from uncomplicated (uUTIs) and complicated (cUTIs) community-onset urinary tract infections in Villa Montes, Bolivia, 2020–2021; Figure S1: Antibiotic susceptibility rates (%) of *E. coli* from community-onset urinary tract infections stratified by gender, Villa Montes, Bolivia, 2020–2021; Figure S2: Antibiotic susceptibility rates (%) of *E. coli* from community-onset urinary tract infections stratified by patient group, in Villa Montes, Bolivia, 2020–2021. UTI, urinary tract infections; uUTI, uncomplicated UTI, cUTI, complicated UTI; Table S6: Co-resistance patterns among MDR *Escherichia coli* (*n* = 182), *Klebsiella pneumoniae* (*n* = 6) and *Proteus mirabilis* (*n* = 3) from community-onset urinary tract infections in Villa Montes, Bolivia, 2020–2021. Table S7: Antibiotic susceptibility rates (%) of CTX-M-producing and CTX-M-negative *E coli* from uncomplicated and complicated community-onset urinary tract infections in Villa Montes, Plurinational State of Bolivia (2020–2021).

## Abbreviations

The following abbreviations are used in this manuscript:

AMR	Antimicrobial resistance
MDR	Multi-drug resistant
UTI	Urinary tract infection
CO	Community onset
uUTI	Uncomplicated urinary tract infection
cUTI	Complicated urinary tract infection
ESBL	Extended-spectrum β-lactamase

## References

[1] Flores-Mireles A.L., Walker J.N., Caparon M., Hultgren S.J. (2015). Urinary Tract Infections: Epidemiology, Mechanisms of Infection and Treatment Options. Nat. Rev. Microbiol..

[2] Levison M.E., Kaye D. (2013). Treatment of Complicated Urinary Tract Infections With an Emphasis on Drug-Resistant Gram-Negative Uropathogens. Curr. Infect. Dis. Rep..

[3] European Association of Urology (EAU) (2022). EAU Guidelines on Urological Infections.

[4] Gupta K., Hooton T.M., Naber K.G., Wullt B., Colgan R., Miller L.G., Moran G.J., Nicolle L.E., Raz R., Schaeffer A.J. (2011). International Clinical Practice Guidelines for the Treatment of Acute Uncomplicated Cystitis and Pyelonephritis in Women: A 2010 Update by the Infectious Diseases Society of America and the European Society for Microbiology and Infectious Diseases. Clin. Infect. Dis..

[5] Al Lawati H., Blair B.M., Larnard J. (2024). Urinary Tract Infections: Core Curriculum 2024. Am. J. Kidney Dis..

[6] Kolman K.B. (2019). Cystitis and Pyelonephritis. Prim. Care Clin. Off. Pract..

[7] Medina M., Castillo-Pino E. (2019). An Introduction to the Epidemiology and Burden of Urinary Tract Infections. Ther. Adv. Urol..

[8] ASUSS (2019). Normas de Diagnóstico y Tratamiento de Nefrología.

[9] Prieto Valtueña J.M., Yuste Ara J.R. (2019). La Clínica y El Laboratorio: Interpretación de Análisis y Pruebas Funcionales. Exploración de Los Síndromes. Cuadro Biológico de Las Enfermedades.

[10] Weinstein M.P., Limbago B., Patel J.B., Mathers A.J., Burnham C.-A., Mazzulli T., Campeau S., Munro S.D., Conville P.S., de Danies M.O.S. (2018). Methods for Dilution Antimicrobial Susceptibility Tests for Bacteria That Grow Aerobically.

[11] Lewis II J.S., Weinstein M.P., Bobenchik A.M., Campeau S., Cullen S.K., Dingle T., Galas M.F., Humphries R.M., Kirn T.J., Limbago B. (2022). Performance Standards for Antimicrobial Susceptibility Testing.

[12] Magiorakos A.-P., Srinivasan A., Carey R.B., Carmeli Y., Falagas M.E., Giske C.G., Harbarth S., Hindler J.F., Kahlmeter G., Olsson-Liljequist B. (2012). Multidrug-Resistant, Extensively Drug-Resistant and Pandrug-Resistant Bacteria: An International Expert Proposal for Interim Standard Definitions for Acquired Resistance. Clin. Microbiol. Infect..

[13] Jarlier V., Nicolas M.-H., Fournier G., Philippon A. (1988). Extended Broad-Spectrum β-Lactamases Conferring Transferable Resistance to Newer β-Lactam Agents in *Enterobacteriaceae*: Hospital Prevalence and Susceptibility Patterns. Clin. Infect. Dis..

[14] Giani T., Antonelli A., Caltagirone M., Mauri C., Nicchi J., Arena F., Nucleo E., Bracco S., Pantosti A., Luzzaro F. (2017). Evolving Beta-Lactamase Epidemiology in *Enterobacteriaceae* from Italian Nationwide Surveillance, October 2013: KPC-Carbapenemase Spreading among Outpatients. Eurosurveillance.

[15] Foglietta G., De Carolis E., Mattana G., Onori M., Agosta M., Niccolai C., Di Pilato V., Rossolini G.M., Sanguinetti M., Perno C.F. (2023). “CORE” a New Assay for Rapid Identification of Klebsiella Pneumoniae COlistin REsistant Strains by MALDI-TOF MS in Positive-Ion Mode. Front. Microbiol..

[16] Coppi M., Cannatelli A., Antonelli A., Baccani I., Di Pilato V., Sennati S., Giani T., Rossolini G.M. (2018). A Simple Phenotypic Method for Screening of MCR-1-Mediated Colistin Resistance. Clin. Microbiol. Infect..

[17] Bartoloni A., Sennati S., Di Maggio T., Mantella A., Riccobono E., Strohmeyer M., Revollo C., Villagran A.L., Pallecchi L., Rossolini G.M. (2016). Antimicrobial Susceptibility and Emerging Resistance Determinants (*bla*_CTX-M_, *RmtB*, *FosA3*) in clinical isolates from Urinary Tract Infections in the Bolivian Chaco. Int. J. Infect. Dis..

[18] Boncompagni S.R., Micieli M., Di Maggio T., Mantella A., Villagrán A.L., Briggesth Miranda T., Revollo C., Poma V., Gamboa H., Spinicci M. (2022). Relevant Increase of CTX-M-Producing *Escherichia coli* Carriage in School-Aged Children from Rural Areas of the Bolivian Chaco in a Three-Year Period. Int. J. Infect. Dis..

[19] Pallecchi L., Malossi M., Mantella A., Gotuzzo E., Trigoso C., Bartoloni A., Paradisi F., Kronvall G., Rossolini G.M. (2004). Detection of CTX-M-Type β-Lactamase Genes in Fecal *Escherichia Coli* Isolates from Healthy Children in Bolivia and Peru. Antimicrob. Agents Chemother..

[20] Wagenlehner F.M.E., Weidner W., Pilatz A., Naber K.G. (2014). Urinary Tract Infections and Bacterial Prostatitis in Men. Curr. Opin. Infect. Dis..

[21] von Wintersdorff C.J.H., Penders J., van Niekerk J.M., Mills N.D., Majumder S., van Alphen L.B., Savelkoul P.H.M., Wolffs P.F.G. (2016). Dissemination of Antimicrobial Resistance in Microbial Ecosystems through Horizontal Gene Transfer. Front. Microbiol..

[22] Giani T., Sennati S., Antonelli A., Di Pilato V., di Maggio T., Mantella A., Niccolai C., Spinicci M., Monasterio J., Castellanos P. (2018). High Prevalence of Carriage of *mcr-1*-Positive Enteric Bacteria among Healthy Children from Rural Communities in the Chaco Region, Bolivia, September to October 2016. Eurosurveillance.

[23] SENASAG (Servicio Nacional de Sanidad Agropecuaria e Inocuidad Alimentaria) (2019). Resolución Administrativa No. 158.

[24] Woodford N., Fagan E.J., Ellington M.J. (2006). Multiplex PCR for Rapid Detection of Genes Encoding CTX-M Extended-Spectrum β-Lactamases. J. Antimicrob. Chemother..

[25] Riccobono E., Di Pilato V., Villagran A.L., Bartoloni A., Rossolini G.M., Pallecchi L. (2014). Complete Sequence of PV404, a Novel IncI1 Plasmid Harbouring BlaCTX-M-14 in an Original Genetic Context. Int. J. Antimicrob. Agents.

[26] Cannatelli A., Giani T., Antonelli A., Principe L., Luzzaro F., Rossolini G.M. (2016). First Detection of the *Mcr-1* Colistin Resistance Gene in Escherichia Coli in Italy. Antimicrob. Agents Chemother..

[27] Di Pilato V., Arena F., Tascini C., Cannatelli A., Henrici De Angelis L., Fortunato S., Giani T., Menichetti F., Rossolini G.M. (2016). *Mcr-1.2*, a New *Mcr* Variant Carried on a Transferable Plasmid from a Colistin-Resistant KPC Carbapenemase-Producing Klebsiella Pneumoniae Strain of Sequence Type 512. Antimicrob. Agents Chemother..

[28] Xavier B.B., Lammens C., Ruhal R., Kumar-Singh S., Butaye P., Goossens H., Malhotra-Kumar S. (2016). Identification of a Novel Plasmid-Mediated Colistin-Resistance Gene, Mcr-2, in Escherichia Coli, Belgium, June 2016. Eurosurveillance.

[29] Roer L., Hansen F., Stegger M., Sönksen U.W., Hasman H., Hammerum A.M. (2017). Novel Mcr-3 Variant, Encoding Mobile Colistin Resistance, in an ST131 Escherichia Coli Isolate from Bloodstream Infection, Denmark, 2014. Eurosurveillance.

[30] Carattoli A., Villa L., Feudi C., Curcio L., Orsini S., Luppi A., Pezzotti G., Magistrali C.F. (2017). Novel Plasmid-Mediated Colistin Resistance Mcr-4 Gene in Salmonella and Escherichia Coli, Italy 2013, Spain and Belgium, 2015 to 2016. Eurosurveillance.

[31] Borowiak M., Hammerl J.A., Deneke C., Fischer J., Szabo I., Malorny B. (2019). Characterization of *Mcr-5* -Harboring *Salmonella Enterica* Subsp. *Enterica* Serovar Typhimurium Isolates from Animal and Food Origin in Germany. Antimicrob. Agents Chemother..

