# Supplementary material for: The Etiology and Antimicrobial Susceptibility of Community-Onset Urinary Tract Infections in a Low-Resource/High-Resistance Area of Latin America
Source: Trop Med Infect Dis. 2025 Feb 27;10(3):64. doi: 10.3390/tropicalmed10030064 (PMC11946812; doi:10.3390/tropicalmed10030064)
Supplement: Supplementary file 1 [file tropicalmed-10-00064-s001.zip › tropicalmed-3412552-supplementary/Supplementary Tables_revised_R1.pdf]

Table S1. Primers and probes used in ESBL-positive *Escherichia coli* from community-onset urinary tract infections in Villa Montes, Bolivia, 2020-2021.

Target genes	Primers/probes	Sequence (5′-3′)[1]	Ref	Positive control
	CTX-M-group-1_FW	AAAAATCACTGCGCCAGTTC	[1]	
bla$_{\text{CTX-M group 1}}$	CTX-M-group-1_REV	AGCTTATTCATCGCCACGTT		*E. coli* V460a (*bla*$_{\text{CTX-M-15}}$) [2]
	CTX-M-group1-P	HEX-TGGCGACGGCAACCGTCACGCTGTT-BHQ-1	[2]	
	CTX-M-group-2_FW	CGACGCTACCCCTGCTATT	[1]	
bla$_{\text{CTX-M group 2}}$	CTX-M-group-2_REV	CCAGCGTCAGATTTTTCAGG		*E. coli* C277a (*bla*$_{\text{CTX-M-2}}$) [2]
	CTX-M-group2-P	FAM-TATTGAGCGTGGGCTCGGTTCTGTCCAG-BHQ-1	[2]	
	CTX-M-group-8/25_ FW	CGATACCACCACGCCATTAG	[2]	
bla$_{\text{CTX-M group 8/25}}$	CTX-M-group-8/25_REV	AACCCACGATGTGGGTAGC	[1]	*E. coli* M26a (*bla*$_{\text{CTX-M-8}}$) [2]
	CTX-M-group8/25-P	CY5-CCTGAATGCTGGCAGCGCCGGTG-BHQ-3	[2]	
	CTX-M-group-9_FW	CAAAGAGAGTGCAACGGATG	[1]	
bla$_{\text{CTX-M group 9}}$	CTX-M-group-9_REV	ATTGGAAAGCGTTCATCACC		*E. coli* V404a (*bla*$_{\text{CTX-M-14}}$) [3]
	CTX-M-group9-P	ROX-CGTGCATTCCGCTGCTGCTGGGCA-BHQ-2	[2]	
	U-CTX-M- FW	ATYRAYACMGCVGATAAYWCGCA		*E. coli* V460a (*bla*$_{\text{CTX-M-15}}$), *E. coli* C277a
All *bla*$_{\text{CTX-M}}$	U-CTX-M- REV	CSGCAATSGGRTTRTAGTTAAC	[2]	(*bla*$_{\text{CTX-M-2}}$), *E. coli* M26a (*bla*$_{\text{CTX-M-8}}$),
	U-CTX-M-P	CY5.5-ATGTGCAGYACCAGTAARGTKATGGC-BHQ-3		*E. coli* V404a (*bla*$_{\text{CTX-M-14}}$)

[1] The amplification program consisted of 35 two-step cycles of 15s at 95 °C and 60s at 60 °C

Table S2. Primers and probes used in colistin-resistant *Escherichia coli* from community-onset urinary tract infections in Villa Montes, Bolivia, 2020-2021.

Target genes	Primers/probes	Sequence (5′-3′)[1]	Ref	Positive control
*mcr-1-*like	Mcr-1-rt-fwd<br>Mcr-1-rt-rev<br>Mcr-1-rt-p	ATCAGCCAAACCTATCCCATC<br>ACACAGGCTTTAGCACATAGC<br>Cy5-GACAATCTCGGCTTTGTGCTGACGATC-BHQ-3	[2]	*E. coli* FI-4531 (*mcr-1*) [4],<br>*K. pneumoniae* KP-6884 *(mcr-1.2)* [5]
mcr-2-like	mcr-2-rt-fwd<br>mcr-2-rt-rev<br>mcr-2-rt-p	AGCGATGGCGGTCTATCCTG<br>CAAAAAACGCCAAATTCATCAAGTC<br>HEX-TGATGGGTGCTATGCTACTGATTGTCG-BHQ-1	[6]	*E. coli* 10505 [7]
mcr-3-like	mcr-3-rt-fwd<br>mcr-3-rt-rev<br>mcr-3-rt-p	CCAATCAAAATGAGGCGTTAGC<br>CACTATAAGTGATGCAAACATCG<br>ROX-GGGCACGAGTTAGAATCCCTTTGAACC-BHQ-2	[8]	*E. coli* SNTR3B6 [9]
mcr-4-like	mcr-4-rt-fwd<br>mcr-4-rt-rev<br>mcr-4-rt-p	CAATTACCAATCTACTGCTGACTG<br>GTAACGCCTTAACTCACTGTTG<br>FAM-CTGCTAATGTTCGTTGGCATTGGGATAG-BHQ-1	[8]	*Salmonella enterica* 10472 10]
mcr-5-like	mcr-5-rt-fwd<br>mcr-5-rt-rev<br>mcr-5-rt-p	GCTGCCTGGATGAAATTCTGC<br>GTGTTCACCAAGGCTTCATGC<br>CY5.5-CAGATGGGTGGTGTCGCAGGTTG-BBQ650	[8]	*S. enterica* 13-SA01718 [11]
mcr-6-like	mcr-6-rt-fwd<br>mcr-6-rt-rev<br>mcr-6-rt-p	ACACAGCATAGTCCTTGGTAC<br>AACAGCACAGTAATCAATAGCATC<br>FAM-CACCAATACTTATCCGATGGCACAAAAC-BHQ-1	[8]	NA
mcr-7-like	mcr-7-rt-fwd<br>mcr-7-rt-rev<br>mcr-7-rt-p	TGGAGACCAACAACAGTGAG<br>CACGAACAGCAGCGAGAAGG<br>HEX-TCGTGCTCTGGTTCCTGCTGAC-BHQ-1	[8]	NA
mcr-8-like	mcr-8-rt-fwd<br>mcr-8-rt-rev<br>mcr-8-rt-p	CATCATACTTATCCGTTCCTTTTC<br>CCACAATTCAATTCTAAAAGCTCC<br>ROX-GTACCAGCAATTATCCTGGCGTTGC-BHQ-2	[8]	NA
mcr-9-like	mcr-9-rt-fwd<br>mcr-9-rt-rev<br>mcr-9-rt-p	ACGACTAAAGTGCCTTTCCAG<br>GATTCATATTCGAGAACATGCAC<br>CY5-CTGGTAAAGGCATTGGTATCACGC-BHQ-3	[8]	NA

[1] The amplification program consisted of 35 two-step cycles of 15s at 95 °C and 60s at 60 °C; NA, not available

Table S3. Etiology of community-onset urinary tract infections (UTI) stratified by patient group, in Villa Montes, Bolivia, 2020-2021. uUTI, uncomplicated UTI; cUTI, complicated UTI.

	**uUTIs (n=213)**		**cUTIs (n=2159)**						**Total UTIs (n=372)**	
			**males (n=56)**		**pregnant (n=40)**		**females (n=63)**			
**Species**	**No.**	**%**	**No.**	**%**	**No.**	**%**			**No.**	**%**
Escherichia coli	184	86.4	43	76.8	38	95	57	90.5	322	86.6
Klebsiella pneumoniae	10	4.7	5	8.9	2	5	3	4.8	20	5.4
Proteus mirabilis	4	1.9	2	3.6	-	-	-	-	6	1.6
Morganella morganii	2	0.9	2	3.6	-	-	-	-	4	1.1
Citrobacter koseri	2	0.9	-	-	-	-	-	-	2	0.5
Staphylococcus saprophyticus	2	0.9	-	-	-	-	-	-	2	0.5
Acinetobacter junii	1	0.5	-	-	-	-	-	-	1	0.3
Acinetobacter pitti	1	0.5	-	-	-	-	-	-	1	0.3
Pseudomonas mendocina	1	0.5	-	-	-	-	-	-	1	0.3
Pseudomonas stutzeri	1	0.5	-	-	-	-	-	-	1	0.3
Enterobacter cloacae	1	0.5	-	-	-	-	-	-	1	0.3
Escherichia vulneris	1	0.5	-	-	-	-	-	-	1	0.3
Klebsiella variicola	1	0.5	-	-	-	-	-	-	1	0.3
Salmonella spp.	1	0.5	-	-	-	-	-	-	1	0.3
Stenotrophomonas maltophilia	1	0.5	-	-	-	-	-	-	1	0.3
Proteus vulgaris	-	-	2	3.6	-	-	-	-	2	0.9
Klebsiella oxytoca	-	-	1	1.8	-	-	-	-	1	0.3
Citrobacter freundii	-	-	1	1.8	-	-	-	-	1	0.3
Enterobacter asburiae	-	-	-	-	-	-	1	1.6	1	0.3
Enterococcus faecalis	-	-	-	-	-	-	1	1.6	1	0.3
Serratia marcescens	-	-	-	-	-	-	1	1.6	1	0.3

Table S4. Discrepancies in bacterial identification between the local laboratory (Bolivia) and the secondary laboratory (Italy).

ID Bolivia	no.	ID Italy (no.)				
Enterobacter aerogenes	16	*Klebsiella pneumoniae* (11)	*E. coli* (2)	*Enterobacter asburiae* (1)	*Stenotrophomonas maltophilia* (1)	*Klebsiella variicola* (1)
Citrobacter spp.	7	*E. coli* (3)	*K. pneumoniae* (1)	*Klebsiella oxytoca* (1)	*Morganella morganii* (1)	*Salmonella* sp. (1)
Serratia marcescens	4	*Pseudomonas stutzeri* (1)	*Pseudomonas mendocina* (1)	*Acinetobacter pittii* (1)	*Acinetobacter junii* (1)	
Escherichia coli	3	*K. pneumoniae* (2)	*E. vulneris* (1)			
Enterobacter sp.	3	*K. pneumoniae* (2)	*E. coli* (1)			
Proteus spp.	2	*E. coli* (1)	*Citrobacter koseri* (1)			

Table S5. Antibiotic susceptibility rates (%) of *E. coli* (n=322), *K. pneumoniae* (n=20) and *Proteus mirabilis* (n=6) from uncomplicated (uUTIs) and complicated (cUTIs) community-onset urinary tract infections in Villa Montes, Bolivia, 2020-2021.

Antibiotic	uUTIs	cUTIs	Total UTIs	*p* value[1]
Ampicillin	18.2	13.2	16.0	0.2
Nalidixic acid	37.4	30.9	34.6	0.2
Trimethoprim-Sulphamethoxazole	40.4	32.9	37.1	0.2
Ciprofloxacin	49.0	38.8	44.6	0.06
Cefotaxime	74.7	70.4	72.9	0.4
Amoxicillin-Clavulanic acid	71.2	63.8	68.0	0.2
Ceftazidime	79.8	75.7	78.0	0.4
Gentamicin	79.3	73.0	76.6	0.2
Nitrofurantoin	90.9	92.1	91.4	0.8
Fosfomycin*	95.7	97.1	96.3	0.6
Colistin*	96.2	97.1	96.6	0.8
Imipenem	100	100	100	1
Meropenem	100	100	100	1

[1], calculated by Fisher's exact test; $p < 0.05$, significant; *, only for *E. coli*

Figure S1. Antibiotic susceptibility rates (%) of *E. coli* from community-onset urinary tract infections stratified by gender, Villa Montes, Bolivia, 2020-2021. *P* value calculated by Fisher's exact test. *, $p < 0.05$; **, $p < 0.01$

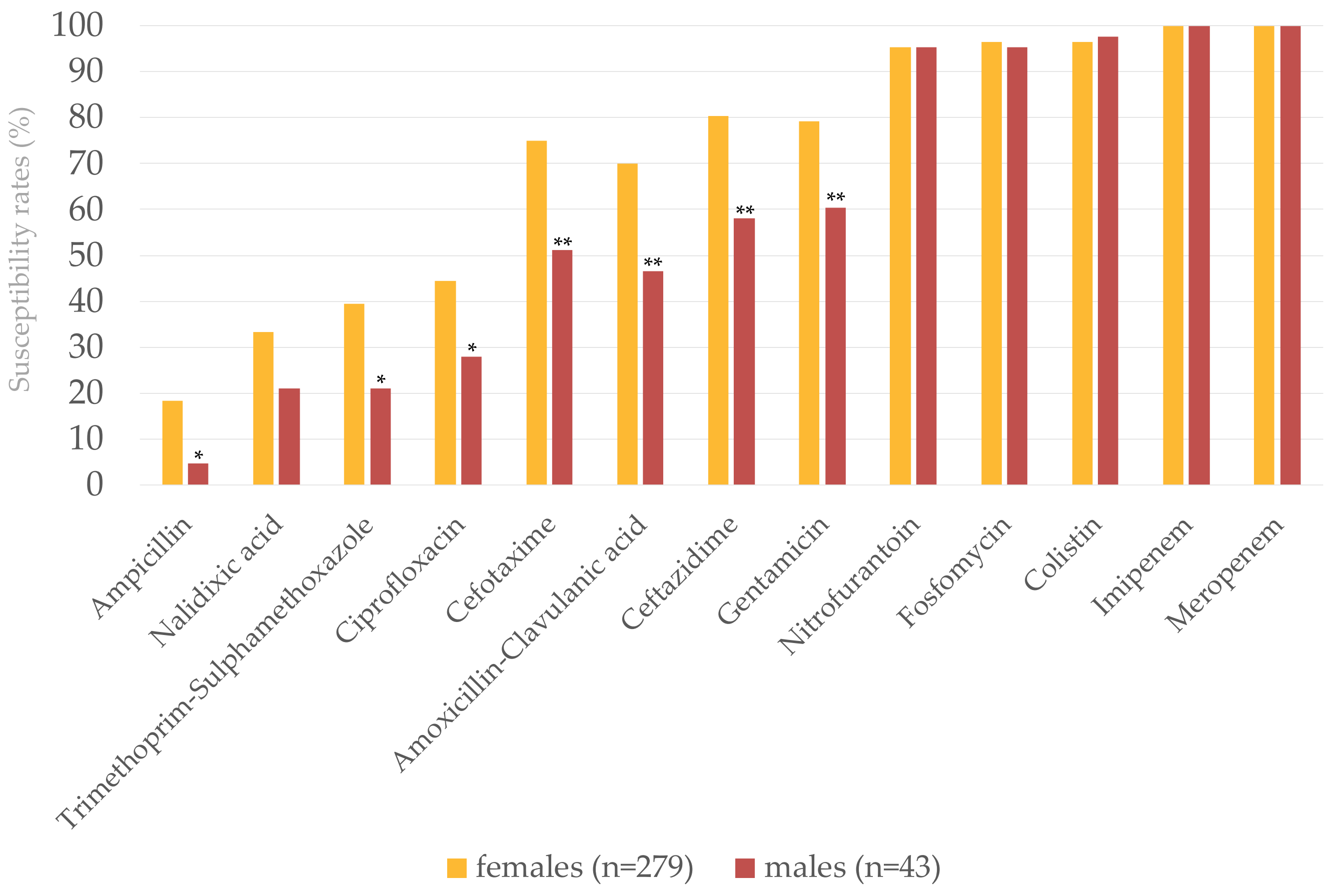

Figure S2. Antibiotic susceptibility rates (%) of *E. coli* from community-onset urinary tract infections stratified by patient group, in Villa Montes, Bolivia, 2020-2021. UTI, urinary tract infections; uUTI, uncomplicated UTI, cUTI, complicated UTI. *P* value calculated by Fisher's exact test. * $p < 0.05$; ** $p < 0.01$

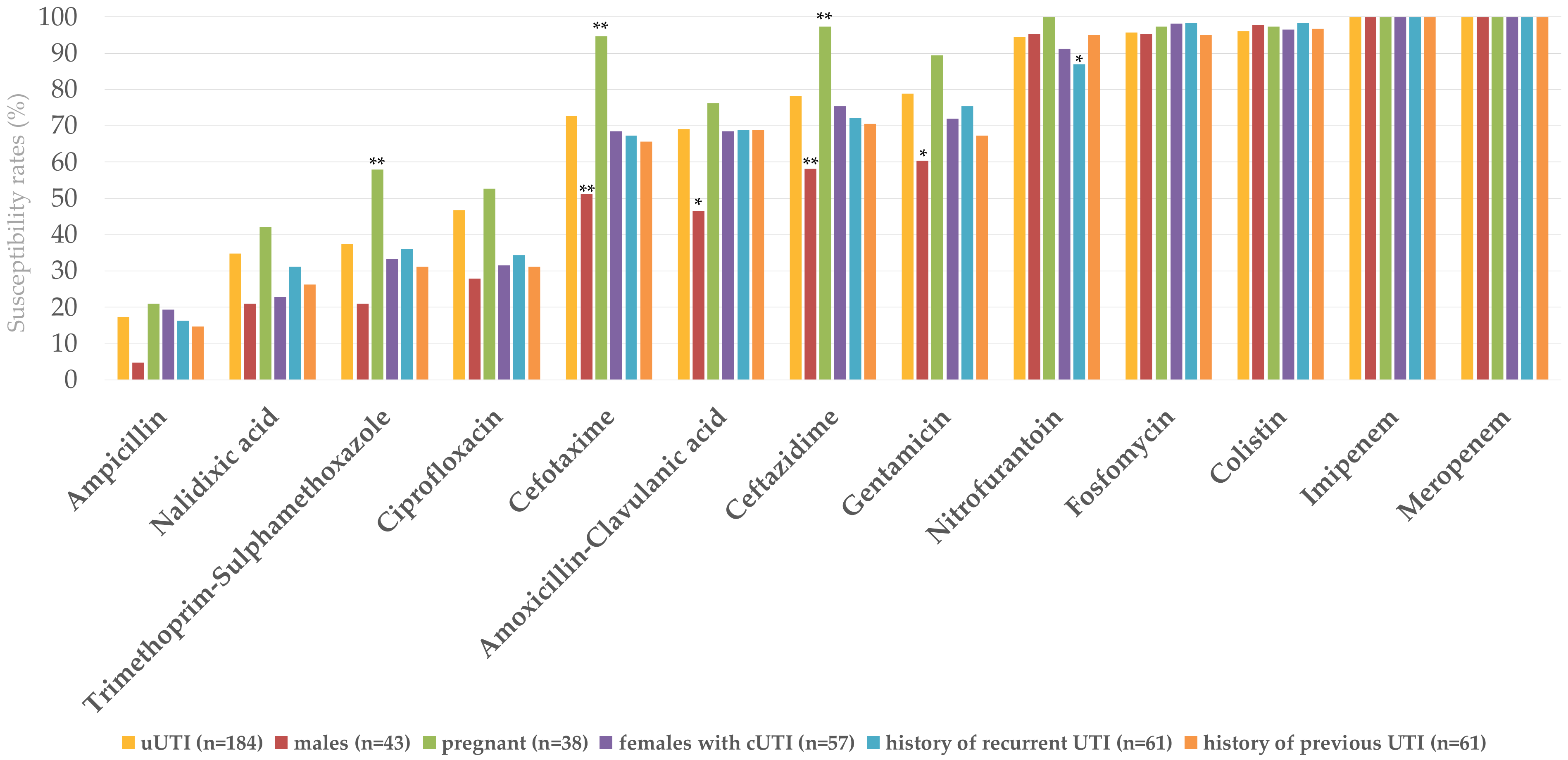

Table S7. Antibiotic susceptibility rates (%) of CTX-M-producing and CTX-M-negative *E. coli* from uncomplicated and complicated community-onset urinary tract infections in Villa Montes, Plurinational State of Bolivia (2020-2021).

Antibiotic	CTX-M-producing *E. coli* (n=86)	CTX-M-negative *E. coli* (n=236)	*p* value[1]
Nalidixic acid	0	44.9	<0.00001
Trimethoprim-Sulphamethoxazole	3.5	57.2	<0.00001
Ciprofloxacin	10.5	44.1	<0.00001
Amoxicillin-Clavulanic acid	50	73.3	0.0002
Gentamicin	58.1	83.5	<0.00001
Nitrofurantoin	91.9	96.6	0.1
Fosfomycin	86.0	97.5	0.0003
Colistin	89.5	98.7	0.0005

[1], calculated by Fisher's exact test; $p < 0.05$, significant

References

1. Woodford, N.; Fagan, E.J.; Ellington, M.J. Multiplex PCR for Rapid Detection of Genes Encoding CTX-M Extended-Spectrum β-Lactamases. *Journal of Antimicrobial Chemotherapy* **2006**, *57*, 154–155, doi:10.1093/jac/dki412.
2. Giani, T.; Antonelli, A.; Caltagirone, M.; Mauri, C.; Nicchi, J.; Arena, F.; Nucleo, E.; Bracco, S.; Pantosti, A.; Luzzaro, F.; et al. Evolving Beta-Lactamase Epidemiology in Enterobacteriaceae from Italian Nationwide Surveillance, October 2013: KPC-Carbapenemase Spreading among Outpatients. *Eurosurveillance* **2017**, *22*, doi:10.2807/1560-7917.ES.2017.22.31.30583.
3. Riccobono, E.; Di Pilato, V.; Villagran, A.L.; Bartoloni, A.; Rossolini, G.M.; Pallecchi, L. Complete Sequence of PV404, a Novel IncI1 Plasmid Harbouring BlaCTX-M-14 in an Original Genetic Context. *Int J Antimicrob Agents* **2014**, *44*, 374–376, doi:10.1016/j.ijantimicag.2014.06.019.
4. Cannatelli, A.; Giani, T.; Antonelli, A.; Principe, L.; Luzzaro, F.; Rossolini, G.M. First Detection of the *Mcr-1* Colistin Resistance Gene in Escherichia Coli in Italy. *Antimicrob Agents Chemother* **2016**, *60*, 3257–3258, doi:10.1128/AAC.00246-16.
5. Di Pilato, V.; Arena, F.; Tascini, C.; Cannatelli, A.; Henrici De Angelis, L.; Fortunato, S.; Giani, T.; Menichetti, F.; Rossolini, G.M. *Mcr-1.2* , a New *Mcr* Variant Carried on a Transferable Plasmid from a Colistin-Resistant KPC Carbapenemase-Producing Klebsiella Pneumoniae Strain of Sequence Type 512. *Antimicrob Agents Chemother* **2016**, *60*, 5612–5615, doi:10.1128/AAC.01075-16.

6. Coppi, M.; Cannatelli, A.; Antonelli, A.; Baccani, I.; Di Pilato, V.; Sennati, S.; Giani, T.; Rossolini, G.M. A Simple Phenotypic Method for Screening of MCR-1-Mediated Colistin Resistance. *Clinical Microbiology and Infection* **2018**, *24*, 201.e1-201.e3, doi:10.1016/j.cmi.2017.08.011.
7. Xavier, B.B.; Lammens, C.; Ruhal, R.; Kumar-Singh, S.; Butaye, P.; Goossens, H.; Malhotra-Kumar, S. Identification of a Novel Plasmid-Mediated Colistin-Resistance Gene, Mcr-2, in Escherichia Coli, Belgium, June 2016. *Eurosurveillance* **2016**, *21*, doi:10.2807/1560-7917.ES.2016.21.27.30280.
8. Foglietta, G.; De Carolis, E.; Mattana, G.; Onori, M.; Agosta, M.; Niccolai, C.; Di Pilato, V.; Rossolini, G.M.; Sanguinetti, M.; Perno, C.F.; et al. “CORE” a New Assay for Rapid Identification of Klebsiella Pneumoniae COlistin REsistant Strains by MALDI-TOF MS in Positive-Ion Mode. *Front Microbiol* **2023**, *14*, doi:10.3389/fmicb.2023.1045289.
9. Roer, L.; Hansen, F.; Stegger, M.; Sönksen, U.W.; Hasman, H.; Hammerum, A.M. Novel Mcr-3 Variant, Encoding Mobile Colistin Resistance, in an ST131 Escherichia Coli Isolate from Bloodstream Infection, Denmark, 2014. *Eurosurveillance* **2017**, *22*, doi:10.2807/1560-7917.ES.2017.22.31.30584.
10. Carattoli, A.; Villa, L.; Feudi, C.; Curcio, L.; Orsini, S.; Luppi, A.; Pezzotti, G.; Magistrali, C.F. Novel Plasmid-Mediated Colistin Resistance Mcr-4 Gene in Salmonella and Escherichia Coli, Italy 2013, Spain and Belgium, 2015 to 2016. *Eurosurveillance* **2017**, *22*, doi:10.2807/1560-7917.ES.2017.22.31.30589.
11. Borowiak, M.; Hammerl, J.A.; Deneke, C.; Fischer, J.; Szabo, I.; Malorny, B. Characterization of *Mcr-5* -Harboring *Salmonella Enterica* Subsp. *Enterica* Serovar Typhimurium Isolates from Animal and Food Origin in Germany. *Antimicrob Agents Chemother* **2019**, *63*, doi:10.1128/AAC.00063-19.
